# Feature Selection for Regression Based on Gamma Test Nested Monte Carlo Tree Search

**DOI:** 10.3390/e23101331

**Published:** 2021-10-12

**Authors:** Ying Li, Guohe Li, Lingun Guo

**Affiliations:** 1 Beijing Key Lab of Petroleum Data Mining, Department of Geophysics, China University of Petroleum, Beijing 102249, China; 2016315014@student.cup.edu.cn (Y.L.); 2019310406@student.cup.edu.cn (L.G.); 2 College of Software, Henan Normal University, Xinxiang 453007, China

**Keywords:** feature selection, regression, nested monte carlo tree search (NMCTS), filter, gamma test, GNMCTS

## Abstract

This paper investigates the nested Monte Carlo tree search (NMCTS) for feature selection on regression tasks. NMCTS starts out with an empty subset and uses search results of lower nesting level simulation. Level 0 is based on random moves until the path reaches the leaf node. In order to accomplish feature selection on the regression task, the Gamma test is introduced to play the role of the reward function at the end of the simulation. The concept Vratio of the Gamma test is also combined with the original UCT-tuned1 and the design of stopping conditions in the selection and simulation phases. The proposed GNMCTS method was tested on seven numeric datasets and compared with six other feature selection methods. It shows better performance than the vanilla MCTS framework and maintains the relevant information in the original feature space. The experimental results demonstrate that GNMCTS is a robust and effective tool for feature selection. It can accomplish the task well in a reasonable computation budget.

## 1. Introduction

Feature selection is a commonly used procedure in data pre-processing. It is further categorized into the filter, wrapper and embedded methods. The filter method generates an optimal feature subset according to a certain evaluation function; it is independent of a succeeded classifier or regressor. Therefore, it can obtain the final result faster. On the contrary, the wrapper method evaluates feature subset according to classifier or regressor result. Thus, it can achieve better performance on the classifier or regressor, but it takes a longer time for the whole process. The embedded method integrates feature selection and model training together. It utilizes learned hypotheses to accomplish feature selection during model-optimized training. In order to achieve a more flexible model combination, the filter method is a good choice.

The Monte Carlo Tree Search (MCTS) method has achieved many states of art performances in the game domain, such as Go [1,2], LOA, Bubble Breaker, SameGame, etc. [3]. These games can be viewed as a large-scale Markov decision process. From this perspective, it can also deal with online planning, route scheduling and combinatorial optimization problems. The success of AlphaGo has had a profound influence on artificial intelligence (AI) approaches. Many reinforcement learning methods were adapted in feature selection problems and achieved satisfactory results. Typically, MCTS for feature selection has developed many fine frameworks [4,5,6]. It can be categorized into the filter or wrapper method depending on the specific framework design. On the one hand, the classifier or regressor results can be directly returned as a reward. On the other hand, evaluation value calculated from certain criteria such as information gain, Fisher’s score, etc., can be used as a reward during iteration. The process can then be considered as a filter method. To be specific, the tree search combines selective strategy and simulation strategy called rollout to obtain the optimal solution. It has a trade-off of exploration versus exploitation, which is also well known as the e–e dilemma. The UCT technique is the most popular way to control the growth of the search tree. The UCB-tuned1 was proposed soon after; this technique adapted well in single-player games, so in this paper, its basic form was also used for feature subset selection. Typically, this paper mainly focused on the regression task. Gamma test was introduced to play the role of the evaluation function. Since MCTS is based on selective sampling and simulation, the result is backpropagated until the episode ends; node values are only updated until then. Speed of convergence and efficient calculation becomes a key. The Gamma test [7,8,9,10] is a non-parametric tool to measure the non-linear relationship between inputs and outputs. It has time complexity O(Mlog M), where M is the number of data points. One run of the Gamma test for thousands of data points usually takes a few seconds. Therefore, the Gamma test can fit this task well. Usually, MCTS takes random moves or follows a simple heuristic strategy during simulation. Nested MCTS (NMCTS) has a stronger performance compared to regular MCTS [11]. NMCTS has beaten MCTS in the deterministic Markov decision process domains such as SameGame, Clickomania. It is natural to expect NMCTS could achieve better performance in feature selection compared to MCTS. NMCTS of higher nesting level uses best search result of lower nesting level as simulation result. A level 1 search corresponds to regular MCTS. Based on the MCTS feature selection method, we proposed the Gamma test nested MCTS method for feature selection in this paper. The main contributions of the study are listed as below:

- The novel method GNMCTS is proposed to solve feature selection on regression tasks, which is less explored in recent researches;
- GNMCTS uses the Gamma test as a reward function, which is easy to implement and takes only a few seconds on a dataset with tens or hundreds of feature dimensions;
- GNMCTS searches the feature space more efficiently through nesting; the two hyper-parameters, nesting level and iteration numbers, are flexible to tune, which can be set to different values on different nesting levels;
- GNMCTS is tested on seven real-world datasets, and the results are compared with the other six feature selection methods based on reinforcement learning. The result shows the superiority of GNMCTS.

The paper is organized as follows: Section 2 briefly reviews the related work. In Section 3, the background methodology on the basic MCTS framework of feature selection is briefly introduced. Section 4 focuses on the GNMCTS method. Given the background of MCTS application in the feature selection domain in Section 4.1, NMCTS was extended to solve the problem in Section 4.2. A revised reward function based on the Gamma test is introduced in Section 4.3. Section 5 mainly compared GNMCTS with other feature selection methods on UCI and WEKA datasets. Conclusions and future work are stated in Section 6.

## 2. Related Work

Feature selection is widely used during data pre-processing. It aims to reduce the data dimensions without losing valuable information and accelerate the succeeded tasks while retaining high accuracy.

The wrapper methods are dependent on the specific classification or regression algorithms. The result of the classifier or regressor acts as an evaluation standard for the candidate feature subsets. Huang [12] proposed a method called FCSVM-RFE for gene detection, where representative genes are ranked by SVM-RFE after gene clustering. Masood [13] proposed to use an incremental search strategy combined with an extreme learning machine classifier. The research of these wrapper methods focused on alleviating time complexity. However, the inherent property of an expensive computation budget is not easy to conquer. Filter methods employ certain measurements such as information gain [14] to evaluate subsets. The main focus lay on improving accuracy, but most researchers pay attention to classification tasks that are not appropriate for regression. Hybrid methods take advantage of both categories. These methods have independent metrics and specific learning algorithms to measure the subsets.

From the perspective of searching strategy, feature selection methods can be categorized into exhaustive, heuristic, meta-search. Exhaustive search is basically impossible to implement on real-world datasets. This leaves the researchers two directions [15] to explore search space: guiding the search process under specific heuristics or using greedy hill-climbing methods. The latter is often simple to implement, such as sequential forward or backward selection (SFS, SBS), the best first search. These methods follow a monotonic behavior of feature selection. The popular heuristics include genetic algorithm (GA), ant colony optimization (ACO) and particle swarm optimization (PSO). Nguyen [16] presented a comprehensive survey on the state-of-the-art works applying swarm intelligence to achieve feature selection in classification, with a focus on the representation and search mechanisms. Sharma [17] conducted a systematic review methodology for synthesis and analysis of one hundred and seventy-six articles. The parameters related to these nature-inspired methods are complex to control and needed to be tuned with great effort. While feature selection based on reinforcement learning method was recently developed with the success of AlphaGo. Fan W. [18] proposed an Interactive Reinforced Feature Selection (IRFS) framework that guides agents by not just self-exploration experience but also diverse external skilled trainers to accelerate learning for feature exploration. The hyper-parameters in these methods are relatively easy to control, and fewer parameters require to be tuned. 

The stopping criteria have a direct influence on the size of the candidate feature subset. It indicates when the search procedure should be stopped. The commonly used criteria include (1) pre-defined number of iterations, (2) pre-defined number of features, (3) difference or improvements between successive iteration steps and (4) judgment by specific evaluation functions. The above criteria do not couple with different methods flexible enough. Automatic stopping criteria should be customized depending on the specific learning algorithms.

In summary, to overcome the problems stated above, the proposed method in this paper focused on the design of the filter feature selection method for the regression task. In order to evaluate the candidate subsets, the Gamma test was used, and NMCTS in game theory was introduced with the merits of easily controllable hyperparameters. The automatic stopping criteria were designed considering the structure characteristic of the search tree and the property of the Gamma test.

## 3. Background Methodology

### 3.1. Basic Procedure of Monte Carlo Tree Search (MCTS) for Feature Selection

**Figure 1:**
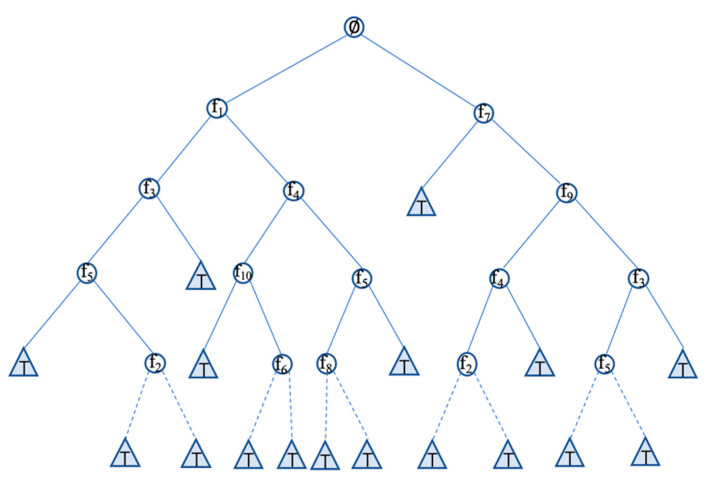
Monte Carlo Tree Search (MCTS).

Feature selection can be regarded as a sequential decision problem. It has many common points with a single-player game that has no opponent. To be specific, the action space and state space are finite and discrete. Given a set of features $F_{All}=\left\{X_{1},X_{2},\ldots ,X_{M}\right\}$, MCTS algorithm will finally return the best action set as the best feature subset $F_{best}$. A brief introduction of MCTS for the feature selection problem is represented in Figure 1. The algorithm can be summarized into the following four basic steps, which are:

(1) Selection: Let $N_{root}$ define the root node where the feature subset is empty (i.e., $F_{root\_sub}\in \emptyset$), starting from $N_{root}$, use some tree policy to gradually descend inside the tree until the path reaches a non-terminal state leaf node $N_{i}$. Choosing an action corresponds to adding a selected feature to the candidate feature subset $F_{sub}=F_{sub}\cup \left\{N_{i}\right\}$, $F_{sub}$ is also used as the state of $N_{i}$;
(2) Expansion: Expand $N_{i}$ until it has no more legal actions that correspond to the case where the remaining feature set is empty (i.e., $F_{All}\backslash F_{sub}=\emptyset$) or pre-conditioned number of expanded children is reached. Then, add expanded children node $N_{j}\;$to $N_{i}$. Initialize $N_{j}\;$with new node state as $F_{sub}=F_{sub}\cup \left\{N_{j}\right\}$, record its parent $N_{i}$. The features already appeared in $F_{sub}$ will no longer be in the legal actions;
(3) Simulation: This procedure is also called a rollout or a playout. In general, starting from the leaf node $N_{i}$, the successive nodes are chosen step by step by some simulation policy until it reaches a terminal state or pre-conditioned computation budget;
(4) Backpropagation: The simulation result is backpropagated through the nodes during the selection phase on the path, and their statistics are updated. The statistics include the visit number of nodes and their values.

The tree search strategy includes two policies. The two policies involved in the selection phase and simulation phase, respectively, are:

(1) Tree policy: It is a strategy to select features. Furthermore, it can be split into two aspects. One is selected during the tree build-up period, and another is the final selection of picking up the best feature sequence $F_{best}$. The former has many variations [19]; the most popular version proposed by Auer et al. is called UCB1, represented by Equation (2), the policy indicates to execute an action with promising potentials which can maximize value in Equation(2),

(1) $$\bar{\mu_{j}}=\frac{Q\left(s,a\right)}{n\left(s,a\right)}\;$$

(2) $$UCB1=\bar{\mu_{j}}+\sqrt{\frac{C_{e}\cdot \ln n}{n_{j}}}$$

where $\bar{\mu_{j}}$ defines average gain of the selected feature, s is the current state which represents $F_{sub}\;$in the feature selection problem, a represents the currently selected action that corresponds to adding a new feature to the current subset. Q(s,a) is an instant reward after adding the new feature to the current subset. n(s,a) defines the number of visits of the current node $n_{i}$, $n_{j}$ defines the number of visits of its children nodes. With the increasing visited number of uncertainty nodes, the asymmetrical growing search tree gradually prefer those nodes that gain a higher exploitation score $\bar{\mu_{j}}$. The confidence interval shrinks with repeated visits. 

To a large degree, how much exploration part accounts for evaluation result relies on the exploration constant $C_{e}$. Aiming at the choice of this parameter, Oleksandr I. Marchenko proposed the MCTS-TSC (tree shape control) method, which used the original depth–width criteria [20]. For the feature selection problem, there is no fixed shape such as depth dominant or width dominant for the search tree. It is implicit in constraining the growing direction of the tree. Considering the complexity of the algorithm and computation budget, $C_{e}$ chosen by trails is a better and easier idea, for those who do not care about the cost may combine the newest technique on pruning. 

For the final feature subset decision, the target is to achieve the highest classification accuracy or minimum regression error, so the tree should choose nodes with the best score record that have been seen so far rather than the average score.

Default policy: It is a strategy to implement a rollout. There are two ways to perform this: either by a uniform random selection policy or by some simple heuristic based on prior domain knowledge. The enhancements on the rollout policy can be found in Cameron B. Browne [21].

The pseudocode for MCTS is listed in Algorithm 1 as follows:
**Algorithm1** MCTS(time_limit,iteration_limit,explorationRate) //explorationRate defines the degree of explorationroot = treeNode(initialState, None) While (time<time_limit & count<iteration_limit) do randomPolicy(state):  while not state.isTerminal():   try:     action = random.choice(state.getPossibleActions())   except IndexError:     raise Exception("Non-terminal state has no possible actions: " + str(state))   state = state.takeAction(action) return state.getReward()   def selectNode(self, node):   while not node.isTerminal:     if node.isFullyExpanded:      node = self.getBestChild(node, self.explorationConstant)     else:      return self.expand(node)   return node def expand(self, node):   actions = node.state.getPossibleActions(node)   for action in actions:     newNode = treeNode(node.state.takeAction(action), node)     node.children[action] = newNode     if len(actions) == len(node.children):      node.isFullyExpanded = True     return newNode  def backpropogate(self, node, reward):   while node is not None:    node.numVisits += 1    node.totalReward += reward     node = node. Parent

### 3.2. Gamma Test

The Gamma test is a non-linear modeling and analysis tool to test the relationship between input and output variables on the numerical dataset. It fits the job of feature subset selection fast enough; the time complexity of the Gamma test is O(MlogM), where *M* is the number of input samples. One single run of the Gamma test takes roughly only a few seconds on a dataset that consists of thousands of instances with hundred features. The Gamma test has already been applied in many industrial and natural resource problems [22,23,24,25]. In the section, a brief introduction of the calculation steps and theory are organized. 

The relationship between input $X_{i}$ and output $y_{i}$ can be represented by a smooth function in the following form:

(3) $$y_{i}=f\left(X_{i}\right)+r$$

where f(X) is the assumed regression model, r is a noise that cannot be explained by f(X). When there is no noise, r is zero.

Define $X_{N\left[i,k\right]}$ as a list of k nearest neighbors of the ith point $X_{i}$ in the input space $\left\{X_{1},X_{2},X_{3},\ldots ,X_{M}\;\right\}$ found by KD tree. p is defined as the number of the nearest neighbors used to calculate statistic Γ. Based on many researches and experiments [26], it is shown that p=10 can obtain better results in a reasonable time.

Define $y_{N\left[i,k\right]}$ as the list of the target value corresponding to the nearest neighbor sequence $X_{N\left[i,k\right]}$. It should be noticed that they are not the list of kth nearest neighbors to the ith point $y_{i}$. Calculate the Euclidean distance between the nearest neighbors and the query point in the input and output space,

(4) $$\delta_{M}\left(k\right)=\frac{1}{M}\overset{M}{\underset{i=1}{\sum }}{\left|X_{N\left[i,k\right]}-X_{i}\right|}^{2}$$

(5) $$\gamma_{M}\left(k\right)=\frac{1}{2M}\overset{M}{\underset{i=1}{\sum }}{\left|y_{N\left[i,k\right]}-y_{i}\right|}^{2}$$

By Equation (3), and the continuity of unknown function f(X), the probability of $\gamma_{M}\left(k\right)\to var\left(\gamma \right)$ as $\delta_{M}\left(k\right)\to 0$. However, it is impossible for $\delta_{M}\left(k\right)$ to reach zero infinitely. Therefore, the limit value $\gamma_{M}\left(k\right)$ that infinitely approximates var(γ) cannot be directly calculated. Finally, by Equation (5),

(6) $$\gamma_{M}\left(k\right)\to var\left(\gamma \right)\;as\;\delta_{M}\left(k\right)\to 0\;$$

the Gamma test assumes that the relationship between the k-neighbor pairs $\delta_{M}\left(k\right),\;\gamma_{M}\left(k\right)$ are approximately linear, and the **slope** is a constant A,

(7) $$\gamma_{M}\left(k\right)=A\delta_{M}\left(k\right)+var\left(\gamma \right)+o\left(\delta_{M}\left(k\right)\right),\;as\;\delta_{M}\left(k\right)\to 0\;\;$$

Based on the above assumptions, the least-squares linear fit is performed on $\left\{(\delta_{M}\left(k\right),\;\gamma_{M}\left(k\right)),\;1<k\leq p\right\}$. Equation (7) can be written as

(8) $$\gamma_{M}\left(k\right)=A\delta_{M}\left(k\right)+\Gamma \;\;$$

The intercept Γ is the estimated noise variance. The evidence of linear progression can be found in the research by Evans [9]. In some cases, Γ value is negative. The first reason is that number of samples is too small, such as under a hundred points, there are no sufficient data points to obtain an accurate outcome. Another reason is the regression model is so smooth that data points can be fully explained. When Γ≤0, it is replaced by |Γ|. Similarly, the case that Γ>var(y) may occur. When this case is true, some pre-process on data, such as abnormal point detection, should be performed. Since the Gamma test can only examine the non-linear relationship between inputs and output, linear regression should also be considered.

## 4. GNMCTS for Feature Selection 

### 4.1. Nested Monte Carlo Tree Search Subsection

**Figure 2:**
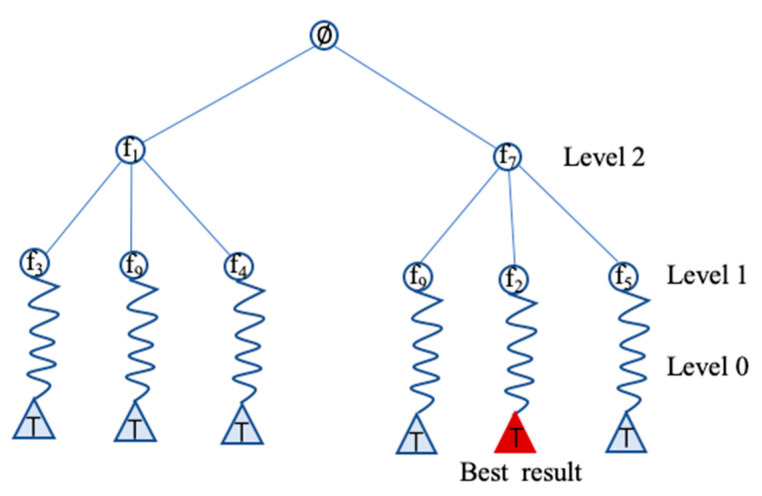
A level 2 NMCTS illustration.

The nested Monte Carlo tree search (NMCTS) was proposed by Hendrik Baier [11]; it was an enhancement work on Nested Monte Carlo Search (NMCS) [21]. The method was tested on many single-player games such as Solitare, SameGame, Bubble Breaker, etc. [27,28,29,30]. It was compared with basic NMCS on different nest levels. NMCTS outperformed regular MCTS on those single-player games, and it can also deal with large Markov decision processes. Therefore, it should adopt the feature selection problem well. NMCTS is different from MCTS in the simulation phase. Selection, expansion and backpropagation phases still remained the same as described in Section 3.1. The NMCTS combined MCTS on a lower base level, leaving itself called recursively on higher nest levels. The techniques of MCTS, such as UCB-tuned1, can also be used in NMCTS. While MCTS uses random feature selection beginning with a given state until reaching a terminal state during rollout, NMCTS uses a heuristic that for every feature selection starting from the given state, and level n search calls level n−1 search result. Then, select the feature with the highest score from level n−1 search. As illustrated in Figure 2, curve lines represent for level 0 search. It is a normal random simulation. Then, level 1 search calls the result of level 0 search and selects the action with the best score. Level 2 search calls level 1 search and selects the feature with the best score.

The best feature sequence is recorded every iteration and compared in case the performance is not improved by adding the new feature. After the computation budget runs out, the best score and sequence are returned. The pseudocode of NMCTS is shown below in Algorithm 2.
**Algorithm 2** NMCTS (startNode, Seq, max_iter, level)  //Seq defines best $F_{sub}$ the tree has found so far best_reward = inf. best_seq = () Current_node = startNode For iteration number in the called level:  While Current_node is not terminal and not fully expanded: Current_node = selection(Current_node) Seq = Current_node.feature_subset If level=1:  While Current_node is not terminal:   Reward, Seq = Random_rollout(Current_node) Else:   Reward, Seq = NMCTS(Current_Node, Seq, max_iter, level-1) Back_propogation(Current_Node, reward) If Reward < best_reward:  best_reward = reward  best_seq = Seq

### 4.2. Gamma Test as Evaluation Function for Regression Task

Next, a simple example was illustrated to show that the Gamma test could be used in feature selection. 

**Figure 3:**
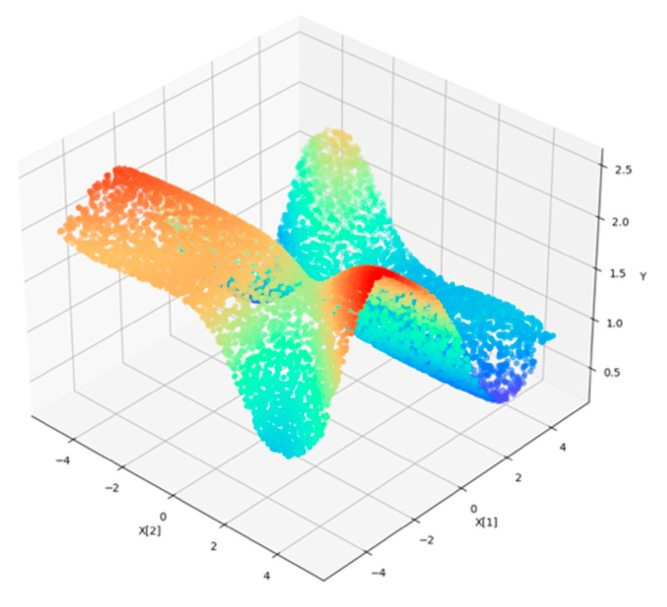
Butterfly 3d projection with X1, X2 and Y.

**Figure 4:**
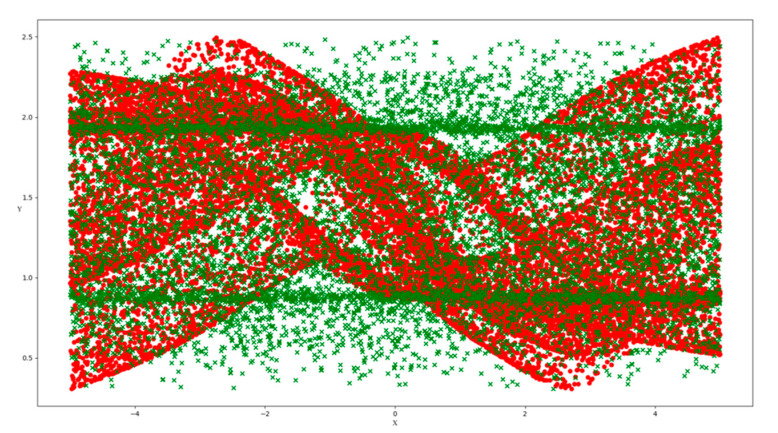
Butterfly scatter plot with X1, X2, I6 and Y.

**Figure 5:**
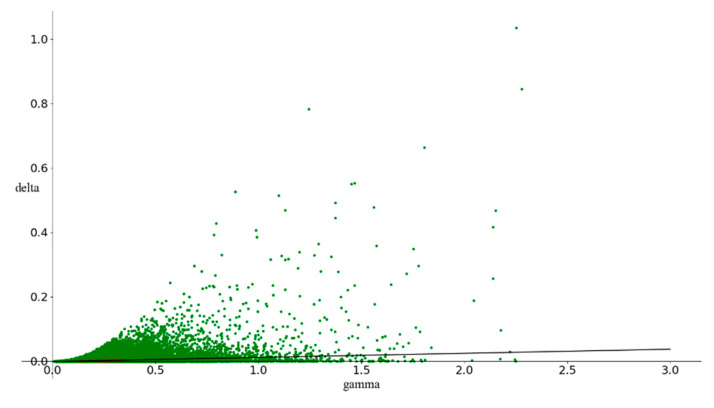
Gamma scatter plot for the smooth function.

The butterfly dataset [31,32] consists of two relevant features, three redundant features and three irrelevant features, which correspond to X1, X2, J3, J4, J5, I6, I7 and I8. In this trial, we generated 10,000 data points with eight features above. Figure 3 illustrates a 3d projection of relevant feature values X1 and X2 on the Y-axis. In Figure 4, an irrelevant feature I6 was added, which was considered as noise. The exhaustive search must traverse 2^8^−1 combinations. As it took only a few seconds, we computed the gamma value for all the possible combinations, and the minimum gamma value should indicate the best relevant feature combination. The combination of the first two features obtained the minimum gamma value of 0.00043 among all cases, which is close to zero, as shown in Figure 5. This validated Gamma test estimated the best feature subset correctly.

### 4.3. Gamma Test Modified Node Selection Policy

In two-player games, the reward is often denoted with {−1,0,1}, representing loss, draw or win. The reward interval of a node falls within [−1,1]. The value of Γ has a large range of variations in different feature subsets. According to Maarten P.D. Schadd [3], there are two solutions; one is scaling the reward back into the interval [−1,1], and the other solution is adding a constant to calculate the reward that would fit the application domain. In the feature selection problem, although the exact maximum Γ value is not known, according to Equation (7), it can be evaluated by the real variance of the target data var(y). For feature selection, a modified UCT version is used. The target is to maximize Equation (9),

(9) $$\mu_{j}+\mathrm{Ce}\cdot \sqrt{\frac{ln\;n}{n_{j}}}+\sqrt{\frac{\Sigma \Gamma^{2}-n_{j}*\mu_{j}^{2}+D}{n_{j}}}\;$$

The left two terms of Equations (9) are the same in Equation (2), the third term contains the sum of squared rollout reward ${\Sigma \Gamma }^{2}$ represents a possible deviation of the child node, it is corrected by the expected results $n_{j}*\mu_{j}^{2}$. Ce and D are constants discussed above aiming at exploring rarely visited nodes. In our experiment, D is set with the value of var(y). Finally, the best feature subset can be found by best policy $\pi^{*}$, which minimizes the Γ value; this can be written in the form of Equation (10).

(10) $$\pi^{*}=\begin{array}{c} \mathrm{argmin}\\ \pi \end{array}\;\Gamma$$

An indicator variable defined:

(11) $$\mathrm{Vratio}\;=\frac{\Gamma }{\mathrm{var}\left(y\right)}$$

Vratio provides a scale-invariant measure; normally, the value is in the range [0, 1]. If the Vratio value is close to zero, then it means the input variable has a strong non-linear relationship with the target. If the Vratio value is close to one, then the prediction target can hardly be explained by input variables; the performance of the regressor is more likely to be a random walk.

To be noted, the filter feature selection method has to generate a subset with a certain number of features. Moreover, the final number of selected features has a direct influence on the result and succeeding computation cost. Romaric [5] proposed to add a stopping feature in the default policy. A stopping feature is chosen with probability $\mathrm{rand}\left(0,1\right)>1-q^{d}$, where *d* is the depth of the current node in the simulation and q is a constant, where q<1. With the growth of the tree, *d* becomes larger, the probability of the stopping feature being selected also becomes bigger. In this paper, the Vratio is considered to replace q, and the modified stopping condition becomes:

(12) $$\mathrm{rand}\left(0,1\right)>{\left(1-V_{\mathrm{ratio}}\right)}^{d}$$

The intuition for the inequality is to achieve a satisfactory regression result with a small number of features. Since Vratio can show the goodness of fitting by the current feature subset, the smaller Vratio is, the smaller the probability of selecting the stopping feature. Then, the tree can further explore the potential path. Otherwise, the larger Vratio is, the sooner the simulation phase ends. The deeper the search tree grows, the bigger probability for the stopping feature to be selected. Another stopping condition takes consideration of the original feature set size of F. For a high dimension feature set, the timing for stopping should be delayed in case feature space is not explored enough. The stopping feature will work if any case in Equation (12) or Equation (13) happens.

(13) $$\mathrm{rand}\left(0,1\right)<\frac{\mathrm{node}.\mathrm{depth}}{\mathrm{size}\left(F\right)}$$

## 5. Experimental Results

This section demonstrates the performance of the NMCTS gamma algorithm on selecting the best feature combination, and the experiments were conducted on seven benchmark datasets. All the experiments were implemented in Python with environment 48 Intel(R) Xeon(R) Silver 4214 CPU 2.20 GHz and 125 GB of RAM.

### 5.1. Datasets

Seven datasets were used for comparison and performance validation. Datasets were taken from two publicly available repositories [33,34], UCI and WEKA. Specific information is shown in Table 1. The feature dimensions and the number of instances varied to gain diversity in characteristics. Both the features and labels are numeric. If datasets contained some ID information, then that column was deleted. The range of labels was listed in the fifth column of Table 1. The Parkinsons_Updrs dataset is composed of a range of biomedical voice measurements from 42 people with early-stage Parkinson’s disease. There are two prediction targets, motor Updrs and total Updrs. To be convenient for comparison, we only considered the total Updrs as a target in the experiments. However, one can calculate the scores, respectively, using the proposed algorithm on multi-output datasets. The Puma32h dataset was synthetically generated from a realistic simulation of the dynamics of a Unimation Puma 560 robot arm. The task is to predict the angular acceleration of one of the robot arm’s links. The Bank32nh was synthetically generated from a simulation of how bank customers choose their banks. Tasks are based on predicting the fraction of bank customers who leave the bank because of full queues. Ailerons addresses a control problem, namely flying an F16 aircraft. The attributes describe the status of the airplane, while the goal is to predict the control action on the ailerons of the aircraft. Pol describes a telecommunication problem in a commercial application. Triazines predicts the activity from the descriptive, structural attributes. Residential building includes construction cost, sale prices, project variables, and economic variables corresponding to real estate single-family residential apartments in Tehran, Iran, and the goal is to predict sale prices.

### 5.2. Experimental Settings

We conducted five-fold cross-validation for all the comparison experiments. The iteration number limit was set to 1000. The corresponding dimension reduction effect and computation time were compared on six datasets of different sizes. The experiment was repeated 20 times then took average values as results. For comparison purposes, the best feature subsets of each feature selection method in Table 2 were tested on the same gradient boosting regressor from the scikit-learn module. Specific parameters of this regressor were: The number of estimators was set to 25, max depth was 4, min samples split was 2, the learning rate was 0.2, the loss was the least square. Before inputting the algorithm, standard normalization was performed for all the datasets. Features with 0 variances that show no contribution to the prediction model were deleted at first.

### 5.3. Comparison Methods and Metrics

We compared the NMCTS gamma algorithm with six state-of-the-art feature selection methods for the regression task listed in Table 2. We mainly focused on feature selection methods using reinforcement algorithms which included temporal difference learning, Q-learning and enhanced MCTS methods. 

A brief introduction of parameter settings related to methods in Table 2 are listed below:

- The objective function of particle swarm optimization (PSO) consists of customized evaluation function results and the feature number reduction ratio. For comparison purposes, the evaluation function’s part in it was substituted by the Gamma test;
- QBSO integrated the Bee Swarm Optimization algorithm with Q learning for solving feature selection tasks. The original algorithm was designed for classification. In the regression case, the fitness of BSO was substituted from the accuracy of the KNN classifier to the mean square error of the KNN regressor. The reward function of Q Learning only differed in minor sign modification from its original paper;
- For MCTS_RreliefF, as the ReliefF algorithm was used to implement classification on multiclass outputs feature selection problem, we changed it into RreliefF algorithm; the other framework in the paper remained the same, including most parameter settings in [38];
- For MCTS with global rave and local rave (MCTS_RAVE), the reward function of MCTS was originally AUC. It was also substituted by the Gamma test;
- For the Temporal Difference learning method, the reward function was also changed into the Gamma test. Learning rate alpha was 0.5, epsilon in the ε-greedy strategy was 0.5. Epsilon decay rate and alpha decay rate were set to 0.995, and the discount parameter was 0.3, parameter b in heuristic was 0.6, stop condition parameter was 3;
- GRNN used the Radical basis function as the kernel. The kernel bandwidth was decided by Silverman’s rule of thumb. Type of the gradient search solver was chosen L-BFGS-B;
- GNMCTS used level 2 nesting search. The iteration number of nesting was set to 10 for level 2 and 100 for level 1. The UCT exploration constant Ce was 0.3. The expansion width of each node was 10. The rest parameters were the same with the MCTS_RAVE method.

The final results were evaluated on seven metrics, including the mean squared error (MSE), mean absolute error (MAE), R-square (R2), explained variance score (EV), dimension reduction (DR) effect, confidence interval and computation time. The expressions of these measurements are as follows:

(14) $$\mathrm{MSE}=\frac{1}{m}\sum_{i=1}^{m}{\left(y_{i}-\hat{y_{i}}\right)}^{2}$$

(15) $$\mathrm{MAE}=\frac{1}{m}\sum_{i=1}^{m}\left|y_{i}-\hat{y_{i}}\right|$$

(16) $$R2=1-\frac{\sum_{i=1}^{m}{\left(y_{i}-\hat{y_{i}}\right)}^{2}}{\sum_{i=1}^{m}{\left(y_{i}-\begin{array}{c} \_\\ y_{i} \end{array}\right)}^{2}}$$

(17) $$\mathrm{EV}=1-\frac{\sum_{i=1}^{m}{\left(y_{i}-\hat{y_{i}}\right)}^{2}-\frac{1}{m}\sum_{i=1}^{m}\left|y_{i}-\hat{y_{i}}\right|}{\sum_{i=1}^{m}{\left(y_{i}-\begin{array}{c} \_\\ y_{i} \end{array}\right)}^{2}}$$

(18) $$P\left(L_{m}<{\hat{y_{i}}}_{i}<U_{m}\right)=\gamma$$

The smaller MSE and MAE are, the more accurate predictions are. On the contrary, the larger R2 and EV are, the more powerful of model predictions are. When the value is close to 1, it indicates the model can perfectly predict all data correctly. When the value is close to 0, it indicates the model performance essentially acts as a baseline model. When the value drops below 0, it indicates the model is worse than the baseline model. This could be the reason why there is no linear or non-linear relationship between inputs and outputs. The difference between R2 and EV lies in the mean value of the residual, i.e., whether $\frac{1}{m}\overset{m}{\underset{i=1}{\sum }}\left|y_{i}-\hat{y_{i}}\right|$ is 0 or not. In Equation (18), γ is a number between 0 and 1, and it was set 0.95 in this paper. $L_{m}$,$\;U_{m}$ are lower and upper confidence bound of variable $y_{i}$.

The dimension reduction ability is represented by Equation (19). The numerator and denominator are the number of selected features and total feature subset, respectively.

(19) $$\mathrm{DR}=1-\frac{\#\mathrm{selected}\;\mathrm{features}}{\mathrm{total}\;\mathrm{features}}$$

### 5.4. Results and Comparisons 

According to the aforementioned parameter settings, experiments were conducted as previously described.

As shown in Table 3a,b, GNMCTS obtained minimum MSE and MAE on Bank32nh and Parkinson’s datasets. On the rest dataset, the results were very close to the best results obtained by GRNN and PSO. GRNN obtained the four best records on triazines, Puma32h, Pol, ailerons and residential building. This could explain why the GRNN method was the wrapper feature selection method. It adjusted neural weights of the hidden layer according to the MSE of regression. Therefore, it has inherent lower MSE and MAE than filter methods, but it cannot deal with a high dimension dataset when the feature number and instance number are large. Additionally, it took a much longer computation time compared with other methods. GRNN failed when calculating the triazines dataset. These were the main problems with GRNN. PSO obtained the smallest MAE and MSE on the triazines dataset but did not perform well in other datasets. GNMCTS was robust and easy to implement. The GNMCTS method obtained better results than MCTS_Rrelieff, PSO, QBSO, MCTS_rave and TD_learning within the same time control. Specifically, GNMCTS outperformed MCTS as expected on four datasets and achieved similar results on Puma32h, Pol and Residential building datasets. This would improve if more iterations were allowed on level 1 or 2 nest level. As the iteration limit was 1000 for both GNMCTS and MCTS, this limited iteration number of GNMCTS on level 1 multiplied by that of level 2 must equal 1000. This would weaken exploration ability on lower-level search space. With the increase in iterations, GNMCTS would finally outperform MCTS. The results of GNMCTS compared with the original dataset without feature selection had slightly improved or maintained the same. 

In Table 3c,d, GNMCTS obtained satisfactory results. Compared with the original dataset without feature selection, it slightly improved on three datasets and held the line on triazines, Pol, Bank32nh, Ailerons. R2 and EV of QBSO and TD learning methods on Puma32h were negative, and the TD learning method also obtained a negative value on Bank32h. These results indicated the models were worse than the baseline model. The baseline model took advantage of mean prediction values, so it was like a conserved guess about the prediction result. This could be due to that the two methods had chosen irrelevant features. GNMCTS, GRNN and MCTS rave methods especially outperform other methods on the Pol dataset. In Table 3e, 95% confidence intervals of the mean value of prediction on seven datasets are presented. As shown in the table, the confidence interval slightly shrunk or remained the same after feature selection compared to the original full feature set. The interval between low and high confidence bound is within a reasonable value.

**Figure 6:**
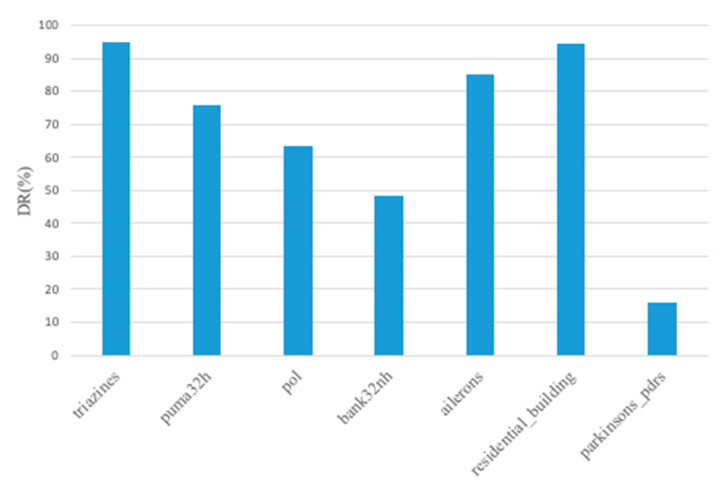
Graphical representation of dimension reduction (DR) achieved by GNMCTS on all datasets.

In order to demonstrate the ability of dimension reduction, the number of selected features in Table 3 was compared with the original dataset. The DR result of GNMCTS is shown in Figure 6. GNMCTS could effectively reduce the feature dimension on most datasets. The Parkinson updrs original dataset only contains 19 columns, so GNMCTS did not need too many iterations to find the optimal solution, but for comparison purpose, we set the iteration number to 1000 which enforce GNMCTS return a relative redundant solution. 

**Figure 7:**
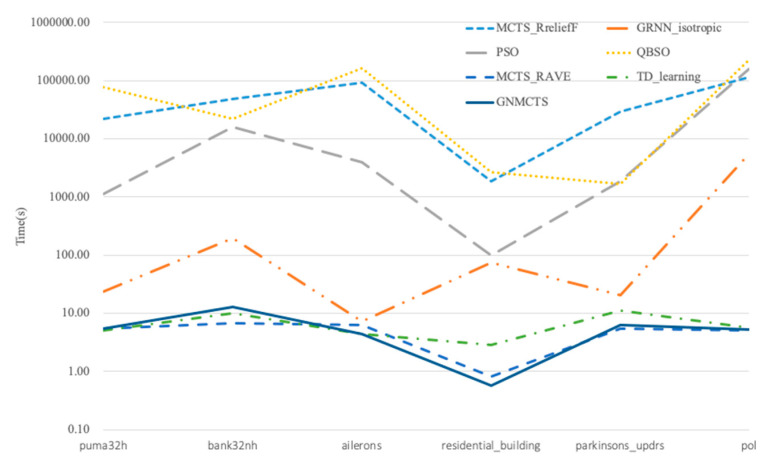
Calculation time comparison illustration of different methods.

The computation times for each method were recorded, as shown in Figure 7. As GRNN failed to predict triazines, the results of this dataset were not shown. With the same iteration number, we can see QBSO was the most time-consuming method. The second most time-consuming method was MCTS_Rrelieff, followed by PSO. The cost of the TD learning method was closed to MCTS RAVE and GNMCTS but was less time-consuming than GRNN.

We also performed the Friedman test on MAE in Table 3b. The Friedman test was used further to compare the generalization of learning methods on different datasets. The *p*-value was 1.8834 × 10^−7^, which was close to 0 and far smaller than 0.05. This means the performances of methods apparently differed from one another. 

## 6. Conclusions

The Monte Carlo Tree Search (MCTS) is a method for searching optimal decisions in a given deterministic environment. It generates an asymmetrical growing tree because of the searching strategy. It combines selectivity and randomness in the search process. The merit of this kind of method is strong learning power without any domain knowledge. This characteristic makes the reinforcement learning method a perfect inspiring player and teacher. It can show some unique ways of solving problems where other methods failed. The proposed method GNMCTS inherits the merits of MCTS and can obtain a better robust result by nesting. Through experimental analysis, GNMCTS obtained satisfactory results compared to other feature methods. It can effectively reduce the feature dimension with a reasonable computation budget. GNMCTS can fit feature selection for regression tasks for data with various dimensions. The Gamma test could indicate how many data points it takes to converge, called the M-test; this could accelerate MCTS greatly. Future work may focus on the revised UCT formulation combined with this M-test and develop an algorithm-based parallelization of NMCTS.

## Figures and Tables

**Table 1:** Benchmark datasets.

No.	Dataset	Instances	Features	Label Range
1	Parkinsons_Updrs	5875	19	[5.0377,39.511]
2	Puma32h	4123	33	[−0.0847,0.0898]
3	Bank32nh	8192	33	[0,0.8197]
4	Ailerons	13,750	41	[−0.0036,0]
5	Pol	15,000	49	[0,100]
6	Triazines	186	61	[0.1,0.9]
7	Residential building	372	109	[50,6800]

**Table 2:** Experimental methods.

Method	Description
PSO	Particle Swarm Optimization based method [35]
QBSO	Q-learning based Bee Swarm optimization method [36,37]
MCTS RreliefF	Improved relief feature selection algorithm based on MCTS [38]
MCTS RAVE	Feature selection as a One-Player Game [39]
FSTD	Feature selections using Temporal Difference [40]
GRNN	General Regression Neural Network [41]

**Table 3:** GNMCTS results compared with other methods on seven datasets.

**(a) MSE:**								
	**MCTS_RreliefF**	**GRNN_isotropic**	**PSO**	**QBSO**	**MCTS_rave**	**TD_learning**	**GNMCTS**	**Original**
triazines	0.2283	--	0.0172	0.2246	0.0183	0.0235	0.0182	0.0169
puma32h	6.60 × 10^−5^	6.50 × 10^−5^	6.90 × 10^−5^	9.07 × 10^−4^	6.60 × 10^−5^	9.22 × 10^−4^	6.70 × 10^−5^	6.90 × 10^−5^
pol	1330.2471	83.5928	1613.6182	1233.6613	99.4774	722.0634	96.1439	84.5149
bank32nh	0.0108	0.0074	0.0072	0.0133	0.0071	0.0151	0.0071	0.0071
ailerons	7.79 × 10^−8^	2.81 × 10^−8^	4.99 × 10^−8^	9.68 × 10^−8^	6.80 × 10^−5^	7.63 × 10^−8^	3.73 × 10^−8^	2.78 × 10^−8^
residential	94,879.2274	51,048.8556	1,107,680	1,098,121	51,887.8991	238,679.153	54,071.2573	54,071.2573
parkisons	18.2702	13.7788	64.3326	64.3017	14.2117	55.4174	13.6362	13.6377
**(b) MAE:**								
**Gradient Boost**	**MCTS _Rrelieff**	**GRNN_isotropic**	**PSO**	**QBSO**	**MCTS _rave**	**TD _learning**	**GNMCTS**	**Original**
triazines	0.1122	--	0.0928	0.1013	0.0951	0.0123	0.0984	0.0906
puma32h	0.0065	0.0064	0.0066	0.0234	0.0065	0.0235	0.0065	0.0066
pol	29.2173	5.3454	34.6598	27.6021	5.9007	18.0749	5.7809	5.4873
bank32nh	0.0732	0.0564	0.0557	0.0828	0.0556	0.0906	0.0552	0.0554
ailerons	1.99 × 10^−4^	1.22 × 10^−4^	1.69 × 10^−4^	2.40 × 10^−4^	6.59 × 10^−3^	2.11 × 10^−4^	1.44 × 10^−4^	1.21 × 10^−4^
residetial	153.4199	109.2683	723.8313	718.452	98.3058	321.5966	117.245	104.9476
parkisons	3.3436	2.8993	6.7796	6.8061	3.0077	6.1205	2.9299	2.9301
**(c) R2:**								
**Gradient Boost**	**MCTS_Rrelieff**	**GRNN_isotropic**	**PSO**	**QBSO**	**MCTS_rave**	**TD_learning**	**GNMCTS**	**Original**
triazines	0.0692	--	0.3046	0.0481	0.2249	0.0399	0.2479	0.3012
puma32h	0.9261	0.9267	0.9229	−0.0187	0.9256	−0.0353	0.925	0.9227
pol	0.2358	0.9519	0.073	0.2913	0.9428	0.5853	0.9449	0.9514
bank32nh	0.2699	0.4962	0.5136	0.103	0.513	−0.0156	0.5111	0.519
ailerons	0.5309	0.8309	0.6997	0.4171	0.9237	0.5411	0.7755	0.833
residetial	0.9343	0.964	0.2285	0.2354	0.9631	0.8338	0.962	0.9574
parkisons	0.7234	0.7912	0.0259	0.0264	0.7846	0.1608	0.7934	0.7934
**(d) EV:**								
**Gradient Boost**	**MCTS_Rrelieff**	**GRNN_isotropic**	**PSO**	**QBSO**	**MCTS_rave**	**TD_learning**	**GNMCTS**	**Original**
triazines	0.075	--	0.3192	0.0831	0.2346	0.0507	0.2646	0.3189
puma32h	0.9262	0.9268	0.923	−0.0165	0.9257	−0.0334	0.925	0.9227
pol	0.236	0.952	0.0732	0.2914	0.9428	0.5854	0.9447	0.9514
bank32nh	0.2701	0.4962	0.5137	0.1043	0.514	−0.0148	0.5111	0.5191
ailerons	0.5312	0.831	0.7001	0.4175	0.9238	0.5415	0.7756	0.833
residential	0.9358	0.9648	0.2346	0.2419	0.9638	0.8362	0.9625	0.9581
parkinsons	0.7237	0.7914	0.0266	0.027	0.7848	0.1611	0.7936	0.7936
**(e) Confidence bound**								
**Gradient Boost**			**GNMCTS**			**Original**		
triazines			[0.6295,0.6691]			[0.6209,0.6968]		
puma32h			[−0.0010,0.0028]			[−0.0010,0.0028]		
pol			[27.5489,30.3289]			[27.5455,30.3560]		
bank32nh			[0.0794,0.0875]			[0.0795,0.0876]		
ailerons			[−8.8193 × 10^−4^,−8.6153 × 10^−4^]			[−8.8513 × 10^−4^,−8.5827 × 10^−4^]		
residential			[1114.2649,1660.1809]			[1114.0922,1649.7011]		
parkinsons			[20.9721,21.6521]			[20.9665,21.6493]		

## Data Availability

Data can be found at https://archive.ics.uci.edu/ml/datasets.php (accessed on 9 June 2021) or https://www.openml.org/home (accessed on 9 June 2021) Codes for methods mentioned in Section 5 can be found at https://github.com/ring0o0o/nmcts.git (accessed on 9 June 2021).
